# A gain-of-function ACTC1 3′UTR mutation that introduces a miR-139-5p target site may be associated with a dominant familial atrial septal defect

**DOI:** 10.1038/srep25404

**Published:** 2016-05-03

**Authors:** Ye Wang, Xinwei Du, Zaiwei Zhou, Jun Jiang, Zhen Zhang, Lincai Ye, Haifa Hong

**Affiliations:** 1 Institute for Pediatric Translational Medicine, Shanghai Children’s Medical Center, Shanghai Jiaotong University School of Medicine, Shanghai, 200127, China; 2 The Cardiothoracic Surgery Department, Shanghai Children’s Medical Center, Shanghai Jiaotong University School of Medicine, Shanghai, 200127, China; 3 Boshi360 Department, WuXi App Tec Co., Ltd., Shanghai, 200131, China; 4 Shanghai Pediatric Congenital Heart Disease Institute, Shanghai Children’s Medical Center, Shanghai Jiaotong University School of Medicine, Shanghai, 200127, China

## Abstract

The ostium secundum atrial septal defect (ASDII) is the most common type of congenital heart disease and is characterized by a left to right shunting of oxygenated blood caused by incomplete closure of the septum secundum. We identified a familial form of isolated ASDII that affects four individuals in a family of five and shows autosomal dominant inheritance. By whole genome sequencing, we discovered a new mutation (c.*1784T > C) in the 3′-untranslated region (3′UTR) of *ACTC1*, which encodes the predominant actin in the embryonic heart. Further analysis demonstrated that the c.*1784T > C mutation results in a new target site for miRNA-139-5p, a microRNA that is involved in cell migration, invasion, and proliferation. Functional analysis demonstrated that the c.*1784T > C mutation specifically downregulates gene expression in a luciferase assay. Additionally, miR-139-5p mimic causes further decrease, whereas miR-139-5p inhibitor can dramatically rescue the decline in gene expression caused by this mutation. These findings suggest that the familial ASDII may be a result of an *ACTC1* 3′UTR gain-of-function mutation caused by the introduction of a new miR-139-5p target site. Our results provide the first evidence of a pathogenic mutation in the *ACTC1* 3′UTR that may be associated with familial isolated ASDII.

Congenital heart disease (CHD) is the most common type of the birth defect. More than 50% of CHDs are caused by sporadic atrial septal defects (ASDs) or ventricular septal defects (VSDs). There are many types of ASDs, which are differentiated from each other by whether or not they involve different structures of the heart. Ostium secundum ASD (ASDII) is the most common type of ASD, comprising 80% of ASDs. Uncorrected ASDII is associated with pulmonary hypertension, right-sided heart failure, flutter or atrial fibrillation, stroke, and Eisenmenger’s syndrome^1^,^2^,^3^.

Recently, several genes have been demonstrated to be associated with sporadic ASD. Pathogenic mutations in *TLL1* and *GATA6* have been identified in families with sporadic ASD^4^,^5^, and mutations in *NKX2.5, GATA4, TBX20, MYH6*, and *TBX5* have been shown to contribute to familial ASD with autosomal dominant inheritance^6^,^7^,^8^,^9^,^10^. Posch *et al*. demonstrated that a gain-of-function *TBX20* mutation (I121M) is pathogenic for ASDII^9^. Furthermore, several pathogenic mutations (p.M123V, p.M178L, p.E101K) in *ACTC1* have been identified in families with isolated ASDII^11^,^12^. ASD5 [OMIM 612794] is one type of ASDII, and it can be produced by mutation in the *ACTC1* gene. *ACTC1*, which encodes alpha cardiac actin on chromosome 15q14, is the only cardiac actin in the embryonic myocardium^13^,^14^. Consistent with the essential role for this protein in mediating heart function, the use of morpholinos to decrease *ACTC1* gene expression in chick embryos results in the occurrence of ASD^11^.

Here, we report a Chinese family with autosomal-dominant isolated ASDII. Whole genome sequencing revealed a mutation in the 3′-untranslated region (3′UTR) of the *ACTC1* genomic region on chromosome 15q14 (c.*1784T > C variant). Screening of this family indicated that the mutation is associated with isolated ASDII with autosomal dominant inheritance. The 3′UTR mutation was functionally analyzed using a Dual-Luciferase Reporter (DLR™) Assay System *in vitro*. The results suggest that the mutation in the 3′UTR of *ACTC1* can reduce the levels of an adjacent luciferase gene. Furthermore, the 3′UTR mutation results in a new target site for miRNA-139-5p, which, according to the database, is expressed in the heart (Http://www.microrna.org). Target validation experiments demonstrate that miR-139-5p inhibitor can dramatically rescue the gene expression decline caused by the c.*1784T > C variant. Collectively, the results suggest that c.*1784T > C may be a pathogenic gain-of-function mutation within the *ACTC1* 3′UTR mutation that accounts for the ASDII within this family. This is the first demonstration of a mutation in the 3′UTR of *ACTC1* that may result in the occurrence of autosomal-dominant isolated ASDII.

## Results

### The clinical characteristics of patients

**Figure 1:**
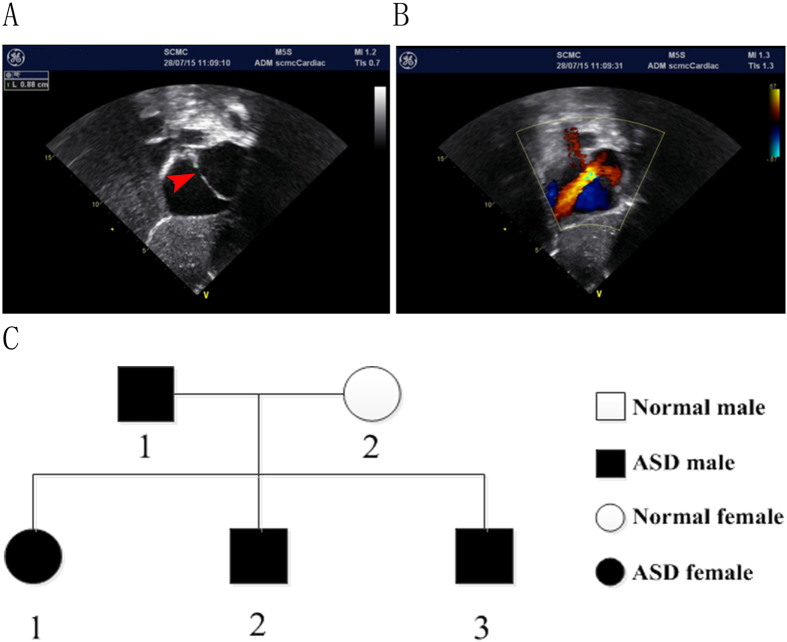
Pedigree with autosomal dominant ostium secundum atrial septal defect (ASDII). (**A**,**B**) Echocardiography of patient II-3. A, the ostium secundum ASD measured 0.88 cm; (**B**) left to right shunting of oxygenated blood. (**C**) Inheritance pattern for the Chinese family with isolated ostium secundum ASD.

We diagnosed a Chinese family with autosomal dominant isolated ostium secundum ASD (Fig. 1). The echocardiography indicated right atrial and ventricle enlargement, normal relaxation and systolic function of the left ventricle, left to right shunting of oxygenated blood, and slightly widened pulmonary arteries (Fig. 1A,B). Four of the five individuals in the family had a similar clinical expression and a diagnosis of ASDII (Fig. 1C), which is characterized by a left to right shunting of oxygenated blood caused by incomplete closure of the septum secundum. Of the four affected individuals in the family, the smallest defect was observed for individual II-3 (measuring 0.88 cm) (Fig. 1A). The cardiomyopathy and other CHDs were not detected in the fifth individual.

### A 3′UTR mutation of *ACTC1* is linked to ostium secundum ASD

To identify the gene defect that accounts for the familial ASD, we performed whole genome sequencing of individual II-3. Coding region variation (including point mutation and indel) and CNV analysis revealed that among the candidate genes that are known to be associated with ASD *(MYH6, TBX20, ACTC1, TLL1, NKX2.5, CITED2, GATA6, CRELD1, TBX5* and *GATA4)*, only *GATA4* had a nonsynonymous mutation (see Supplementary Table S1). However, the mutation in *GATA4* (NM_002052:exon2:c.C487T:p.P163S) was not co-segregated by Sanger sequencing (see Supplementary Fig. S1), which ruled out *GATA4* as an explanation for the occurrence of the ASD.

**Figure 2:**
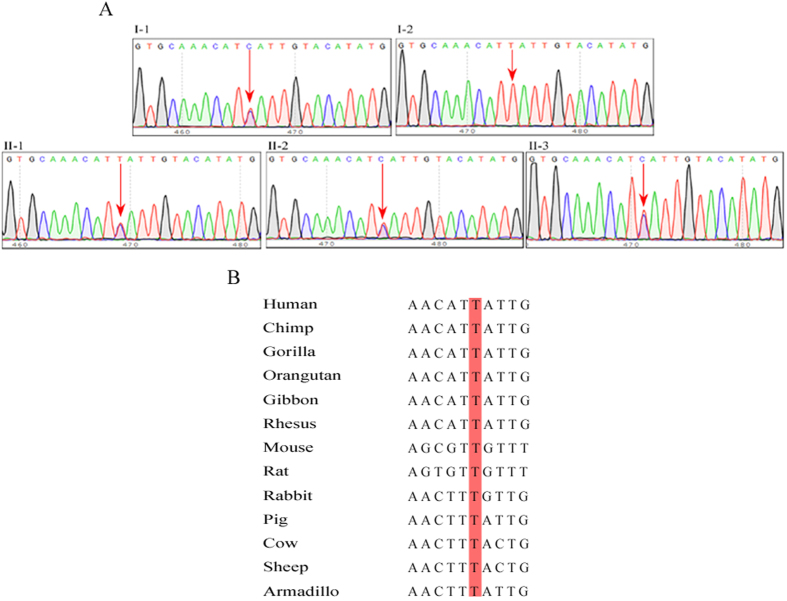
(**A**) Cosegregation was confirmed by Sanger sequencing. I-1, II-1, II-2, and II-3 were ASDII patients and carried a c.*1784T > C mutation in the 3′UTR of *ACTC1*; I-2 is an unaffected family member and did not carry the mutation. (**B**) The conservation of thymine (T) at position 1784 in the *ACTC1* 3′UTR, which are highly conserved across most vertebrate species.

The UTR regions of these 10 genes were reanalyzed after the coding region analyses and we found a mutation in the 3′UTR of *ACTC1* (c.*1784T > C), which encodes the predominant actin in the embryonic heart. The 3′UTR mutation of *ACTC1* was also confirmed by Sanger sequencing. Analysis of the family members revealed that the 3′UTR mutation of *ACTC1* co-segregated in all affected individuals (II-1, 2, 3 and I-1) and the unaffected family member (I-2) did not carry the mutation (Fig. 2A). Furthermore, we searched public databases, including 1000 Genomes, the Exome sequencing project (ESP), and dbSNP, and found that the 3′UTR mutation of *ACTC1* (c.*1784T > C) was not reported. The thymine at position 1784 in the 3′UTR of *ACTC1* is highly conserved in diverse species, including monkeys and apes, rats, mice, rabbits, pigs, and armadillos (Fig. 2B). Collectively, our findings suggest that the c.*1784T > C mutation of *ACTC1* follows an autosomal dominant pattern of inheritance and may be associated with the occurrence of ASD.

### Identification of a microRNA target in the *ACTC1* 3′UTR mutant

**Figure 3:**
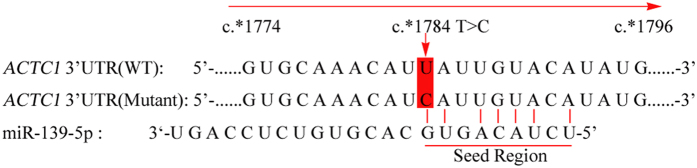
Introduce a miR-139-5p target site at position 1784 bp in the c.*1784T > C mutant. Using Segal prediction software (8-mer matched, allowing one mismatch and one G: U), a miR-139-5p target site was identified in the c.*1784T > C mutant, but not the wild-type sequence.

Because the novel mutation identified in this study resides within the 3′UTR of *ACTC1* immediately following the stop codon, we postulated that the sequence may have a regulatory function in gene expression. To assess this possibility, we used miRNA target prediction software to scan the entire 2316 bp 3′UTR of *ACTC1*, as well as the mutant sequence at position 1784 bp (c.*1784T > C). Using Segal prediction software (8-mer matched, allowing one mismatch and one G: U)^15^, a miR-139-5p target site was identified in the c.*1784T > C mutant, but not the wild-type sequence. These findings suggest that the mutation in the 3′UTR of *ACTC1* may have resulted in a new miR-139-5p binding site (Fig. 3).

**Figure 4:**
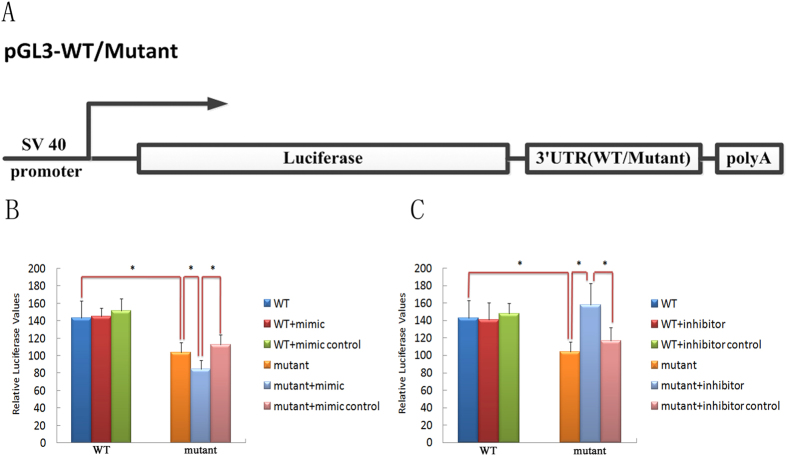
miR-139-5p directs repression of a luciferase reporter gene bearing the mutant 3′UTR segment from *ACTC1*. (**A**) construction of luciferase report plasmids. The wild type and mutant 3′UTR were cloned into the pGL3 plasmid (Promega) downstream of the luciferase gene. (**B**) miR-139-5p mimic (mimic) or control mimic (mimic control) was co-transfected with pGL3-WT (WT) or pGL3-mutant (mutant) reporter into HEK293FT cells. TK-Renilla luciferase plasmid was also co-transfected for normalization. (**C**) miR-139-5p inhibitor (inhibitor) or control inhibitor (inhibitor control) was co-transfected with pGL3-wild (wild) or pGL3-mutant (mutant) reporter into HEK293FT cells. TK-Renilla luciferase plasmid was also co-transfected for normalization. Eighteen replicates were performed (three independent experiments, six cultures each). After normalizing to Renilla activity, Relative luciferase values from pGL3-mutant plasmid were compared to values from pGL3-WT plasmid, and the values from pGL3-mutant plasmid plus mimic/inhibitor was compared to values from pGL3-mutant plasmid plus mimics/inhibitor control and pGL3-mutant plasmid. (*p < 0.05; one-way ANOVA test, SNK procedure served as a multiple comparison procedures).

To determine whether the 3′UTR mutation affects gene expression, we cloned wild-type and mutant 3′UTR segments into an SV-40 driven luciferase expression plasmid (pGL3) essentially as described^16^ (Fig. 4A). The pGL3 plasmids were transfected with 20 ng of Renilla luciferase plasmid to normalize for transfection efficiency. Furthermore, to directly assess whether miR-139-5p can regulate the activity of the mutated 3′UTR, we design a specific miR-139-5p mimic and inhibitor and a non-specific control mimic and inhibitor from Guangzhou RiboBio Co., Ltd.

The results revealed that the mutant UTR caused ~30% decrease in gene expression (Fig. 4B,C, mutant vs. WT, p < 0.05). Moreover, the miR-139-5p mimic caused a further (42%) decline in the activity of the mutant construct (Fig. 4B, mutant+mimic vs. mutant+mimic control, p < 0.05), without any significant effect on the activity of the wild-type construct (WT+mimic vs. WT+mimic control, p > 0.05). Conversely, the miR-139-5p inhibitor reversed the decline in activity of the mutant (Fig. 4C, mutant+inhibitor vs. mutant+inhibitor control, p < 0.05). These results suggest that overexpression of miR-139-5p further downregulates the expression of the mutant UTR and that miR-139-5p inhibitor can completely rescue the decline in gene expression caused by the mutant UTR. Collectively, our results indicate that the mutant *ACTC1* 3′UTR constitutes a target site for miR-139-5p, which can downregulate gene expression. Because reducing ACTC1 levels can result in ASD^11^, it is likely that this gain-of-function mutation explains the pathogenesis of the disease.

## Discussion

In previous studies, many genes have been associated with the occurrence of CHD, and most CHDs are, in fact, associated with coordinated mutations in multiple genes. ASD is a relatively common CHD, comprising approximately 10% of CHDs. Recently, several genes have been confirmed to be involved in the occurrence of ASD. *TLL1* and *GATA6* have been identified as pathogenic mutations in sporadic ASD^4^,^5^, while *NKX2.5, GATA4, TBX20, MYH6*, and *TBX5* contribute to ASD in families with autosomal dominant inheritance^6^,^7^,^8^,^9^,^10^. With the exception of *GATA4*, all of these ASD-associated genes have a common characteristic: the mutations of these genes are in coding region. Reamon-Buettner *et al*. showed that c.*119A > T in the 3′UTR of *GATA4* is associated with ASDII in a family, but that there is no cosegregation with disease^17^. Therefore, up to now, no mutations that are regulated by autosomal dominant inheritance in the 3′UTR of genes have been reported to be involved in familial ASDII.

In our study, we identified a family with autosomal dominant inheritance of isolated ASDII that may be associated with a novel mutation in the 3′UTR of *ACTC1. ACTC1* encodes alpha cardiac actin, which is the only cardiac actin in embryonic myocardium^13^,^14^. Mammals, including mice, cannot survive more than 2 weeks without *ACTC1*^18^. Several studies have determined that *ACTC1* is involved in the occurrence of ASDII. Mutations (p.M123V, p.M178L, p.E101K) in *ACTC1* have been shown to result in ASDII^11^,^12^. In a recent study, Jiang *et al*. reported that 78.8 percent of patients with sporadic CHD (gestational age 18 weeks-49 months) showed significantly reduced *ACTC1* expression. This included about 60% decrease in 26 out of 33 cases (11 out of 15 cases of ASD [atrial septal defect]; 4 out of 4 cases of TOF [tetralogy of Fallot]; 7 out of 10 cases of VSD [ventricular septal defect]; and 4 out of 4 cases of AVSD [atrio-ventricular septal defect])^19^. Matsson *et al*. also identified a 17-bp deletion of *ACTC1* in a case of ASD that predicted a nonfunctional protein^11^. Furthermore, knockdown of *ACTC1* via morpholino antisense nucleotides causes delayed looping and incomplete interatrial septum formation in chick embryos^11^. These data suggest that downregulation of the level of *ACTC1* may lead to ASDII.

In the present study, a new mutation in the 3′UTR of *ACTC1* (c.*1784T > C) was identified by whole genome sequencing for one individual, and was further demonstrated to cosegregate with ASDII for four other members of the family that were tested. By searching the available public database including microRNA.org and Gene Expression Omnibus (GEO), we confirmed that miR-139-5p is expressed in human heart tissue (Http://www.microrna.org, and GSM995302). This indicated that miR-139-5p and *ACTC1* are expressed in the same tissue. The results suggest that the c.*1784T > C might be a pathogenic mutation that is inherited in an autosomal dominant manner. The c.*1784T > C variant is localized in the 3′UTR of *ACTC1*. MicroRNA response elements are known to play important roles in the regulation of mRNA transcripts via their 3′UTRs, with regulatory functions in translation, stability, and localization^20^,^21^. Recent studies have shown that cardiac diseases are influenced by the overexpression or suppression of several specific miRNAs in mice, including miR-1, miR-1-2, miR-195, miR-208, and miR-133^22^,^23^,^24^,^25^,^26^,^27^,^28^,^29^,^30^,^31^,^32^. These miRNAs play crucial roles in cardiomyocyte proliferation, construction, ventricular integrity, and cardiac rhythm maintenance. At the 1784 bp region of the wild-type *ACTC1* 3′UTR, no microRNA binding sites are predicted. However, the c.*1784T > C variant is predicted to create a *de novo* binding site for miR-139-5p. Though miR-139-5p has not been reported to be involved in heart development, it is thought to be involved in the cell migration, invasion, and proliferation in tumors^33^,^34^. Based on the pattern of cosegregation within the family, the acquired binding site is likely to be associated with the occurrence of ASDII.

The 3′UTR of *ACTC1* is about 2316 bp in length. We confirmed that *ACTC1* does not have any alternative 3′UTR or poly-A site by screening NCBI Reference Sequences. There are many predicted microRNA binding sites including the miR-139-5p site in the *ACTC1* 3′UTR (see Supplementary Tables S2 and S3). However, in this study, using an *in vitro* Dual-Luciferase Reporter (DLR™) Assay System, we confirmed that the c.*1784T > C variant in the *ACTC1* 3′UTR results in 30% decreased gene expression. Furthermore, the overexpression of miR-139-5p by the addition of 100 nM miR-139-5p mimic can cause 12% additional decrease in the gene expression level for the mutant, but has no effect on wild-type UTR expression. Conversely, the miR-139-5p inhibitor (150 nM) can completely rescue the gene expression decline caused by the c.*1784T > C variant. These findings strongly suggest that the *ACTC1* 3′UTR mutation might be a new pathogenic gain-of-function mutation that causes *ACTC1* to become a target gene for miR-139-5p, which can downregulate its gene expression level.

In summary, we identified a novel mutation in the *ACTC1* gene by whole genome sequencing. Specially, the mutation was discovered in the 3′UTR of *ACTC1* and was shown to cosegregate with ASDII in an autosomal-dominant manner. miR-139-5p was shown to target the acquired binding site and lead to a reduction in gene expression. These findings are unique in that they suggest a novel mechanism of familial CHD and a new role for miR-139-5p. Though this is the first gain-of-function mutation of a microRNA target site that has been suggested to be involved in CHD, it is likely that additional microRNAs may play key roles in cardiac morphogenesis. Finally, because ostium secundum ASD is the most common type of CHDs, and families with ASDII comprise approximately 10% of all ASD cases^35^, it is worth further exploring the role of *ACTC1* as a candidate gene for cardiac pathogenesis in other families with inherited ASD.

## Materials and Methods

### Patients and sample collection

The ASDII family with autosomal dominant inheritance was diagnosed in the Shanghai Children’s Medical Center by echocardiography. DNA was extracted using the Qiagen blood extract kit. Patient II-3 consented to whole genome sequencing and the use of echocardiographic images. All affected individuals in this study have been corrected through surgical operation. All procedures followed the declaration of Helsinki, and were approved by the Animal Welfare and Human Studies Committee at Shanghai Jiaotong University School of Medicine. Parental written informed consents were obtained prior to the initiation of the study.

### Whole genome sequencing

Whole genome sequencing for patient II-3 was performed by Shanghai WuXiAppTec Co., Ltd on an Illumina HiSeq X Ten system. Picard Collect Wgs Metrics was used to calculate the depth and coverage for whole genome sequencing. The mean depth of whole genome was 38.7, and above 5×, 10×, 20× depth of whole genome coverage corresponded to 98.2%, 95.9% and 75.8%, respectively. DNA concentrations were measured with the NanoDrop 2000 (Thermo Fisher Scientific) and sheared with the S220 Sonicator (Covaris) to an average target size of 500–600 bp. Fragmented DNA was purified using Sample Purification Beads (Illumina). Adapter-ligated libraries were prepared with TruSeqNano DNA Sample Prep kits (Illumina) according to the manufacturer’s protocol. The DNA concentrations of the resulting sequencing libraries were measured with the Qubit 2.0 fluorometer DNA HS Assay (Thermo Fisher Scientific). Quantities and sizes of the resulting sequencing libraries were analyzed using an Agilent BioAnalyzer 2100 (Agilent). The libraries were used in cluster formation on an Illumina cBOT cluster generation system with HiSeq X HD PE Cluster Kits (Illumina). Paired-end sequencing was performed using the Illumina HiSeq X according to the manufacturer’s protocol for 2 × 150 paired-end sequencing. Post-quality control reads were aligned to the reference human genome.

### Mutation analysis and cosegregation analysis by Sanger sequencing

The 3′UTR mutation was assessed through Web prediction software including Segal Lab, polyphen-2, SNP database, and miRNA database. The 3′UTR mutations in all family members were confirmed by Sanger sequencing for cosegregation analysis. The *ACTC1* 3′UTR primers were as follows: forward primer: 5′-ACCAAAGACGTAGGCACTATCCATGTCTCA-3′; reverse primer: 5′-TTGATGAGGATTCAAGACACA-3′.

### Cell culture and Luciferase reporter system

HEK293FT cell were cultured in DMEM (Invitrogen, 11965-092) with 10% FBS (Hyclone) and seeded in 24-well plate. The expression level of miR-139-5p in the HEK293 cells was tested using real-time PCR. The results indicated that miR-139-5p is present in the HEK293FT cells (see Supplementary Fig. S2).

The 3′UTR of *ACTC1* is about 2316 bp in length, and the mutation position is at 1784 bp. The entire 3′UTR was cloned. Wild-type and mutant UTR segments were inserted into the pGL3-control plasmid (E1742, Promega) with an SV-40 promoter, designated pGL3-WT and pGL3-mutant. The miR-139-5p mimic and inhibitor were designed by Guangzhou RiboBio Co., Ltd. Transfections were performed according to the Fugen HD (Progema, E2311) procedure. HEK293FT were seeded in 24-well plate, the next day 100 nM of miRNA mimic or 150 nM of miRNA inhibitor was transfected together with 20 ng TK-Renilla and 2 μg pGL3-WT/pGL3-mutant. Luciferase activity was read using a Synergy 2 (BioTek Co., Ltd.) device and Gene 5 software. The luciferase values were normalized to the activity of Renilla. Relative luciferase values from pGL3-mutant plasmid were compared to values from pGL3-WT plasmid, and the values from pGL3-mutant plasmid plus mimic/inhibitor were compared to values from pGL3-mutant plasmid plus mimics/inhibitor control and pGL3-mutant plasmid. Statistical analysis was performed using one-way ANOVA testing, and SNK for post hoc test (*p < *0.05*).

## Additional Information

**How to cite this article**: Wang, Y. *et al*. A gain-of-function ACTC1 3′UTR mutation that introduces a miR-139-5p target site may be associated with a dominant familial atrial septal defect. *Sci. Rep*. **6**, 25404; doi: 10.1038/srep25404 (2016).

## Supplementary Material

Supplementary Information

Supplementary Table S2

Supplementary Table S3
