# Supplementary material for: A gain-of-function ACTC1 3′UTR mutation that introduces a miR-139-5p target site may be associated with a dominant familial atrial septal defect
Source: Sci Rep. 2016 May 3;6:25404. doi: 10.1038/srep25404 (PMC4853704; doi:10.1038/srep25404)
Supplement: Supplementary Information [file srep25404-s1.pdf]

A gain-of-function ACTC1 3'UTR mutation that introduces a miR-139-5p target site may be associated with a dominant familial atrial septal defect

Ye Wang[1#], Xinwei Du[3#], Zaiwei Zhou[4], Jun Jiang[2], Zhen Zhang[1, 2], Lincai Ye[1*], Haifa Hong[2, 3*]

#, Contribute equally: **Ye Wang**, wangye@scmc.com.cn; **Xinwei Du**, duxinwei@scmc.com.cn

*, Corresponding authors: **Lincai Ye,** ylc717@163.com**; Haifa Hong,** hhfsmallboat@163.com

[1]Institute for Pediatric Translational Medicine, Shanghai Children's Medical Center, Shanghai Jiaotong University School of Medicine, Shanghai, 200127, China

[2]Shanghai Pediatric Congenital Heart Disease Institute, Shanghai Children's Medical Center, Shanghai Jiaotong University School of Medicine, Shanghai, 200127, China

[3]The Cardiothoracic Surgery Department, Shanghai Children's Medical Center, Shanghai Jiaotong University School of Medicine, Shanghai, 200127, China

[4]Boshi360 Department, WuXi App Tec Co., Ltd., Shanghai, 200131, China

Fig.S1

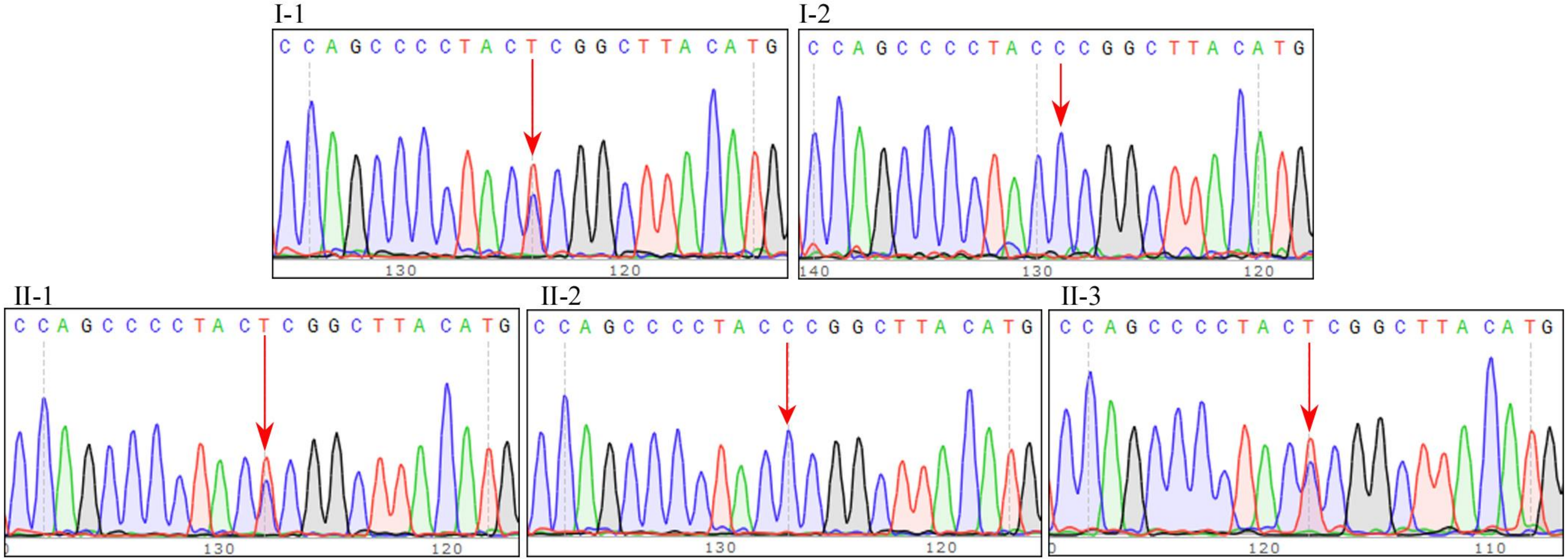

Fig.S1 *GATA4* (NM_002052:exon2:c.C487T:p.P163S) is not Cosegregated with the familial ASD. I-1, II-1, II-2, and II-3 were ASD patients. II-2 is affected individual but not carry the *GATA4* (NM_002052:exon2:c.C487T:p.P163S) mutation.

Fig.S2

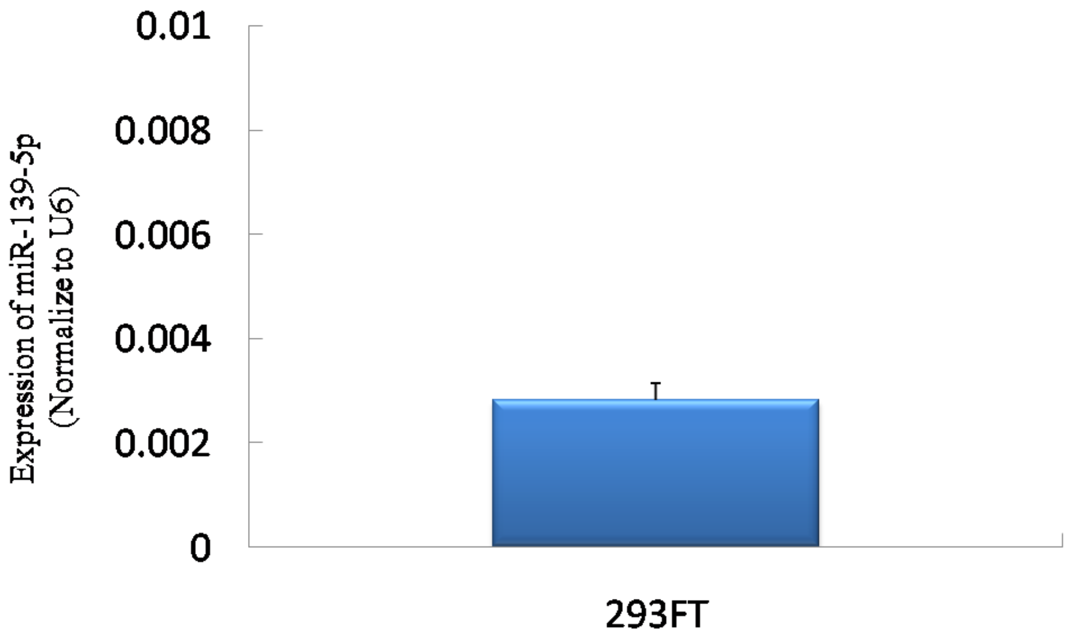

Fig.S2 Detected the expression level of miR-139-5p in HEK293FT cell by real time PCR. Using the Bulge-Loop miRNA qRT-PCR Starter Kit (Guangzhou RiboBio Co., Ltd.), we detected the expression level of the mir-139-5p. The data was normalized to U6.

Table S1

##reference=file:///wgc/prod/database_NGS/hg19fastadatabase/ucsc.hg19.fasta											
#Chromo	Position	Ref	Alt	avsnp142	Func.refGene	Gene.refGene	GeneDetail.refGene	ExonicFunc.refGene	AAChange.refGene	esp6500s	1000g2014oc
Chr3	9976159	A	G	rs279552	exonic	CRELD1	NM_015513:exon1:c.A37G<br>NM_001031717:exon2:c.A37G<br>NM_001077415:exon2:c.A37G	nonsynonymous SNV	CRELD1:NM_015513:exon1:c.A37G:p.M13V<br>CRELD1:NM_001031717:exon2:c.A37G:p.M13V<br>CRELD1:NM_001077415:exon2:c.A37G:p.M13V	0.9915	0.995607
Chr3	9985656	C	T	rs3774207	exonic	CRELD1	NM_001031717:exon11:c.C1119T	synonymous SNV	CRELD1:NM_001031717:exon11:c.C1119T:p.H	0.4055	0.379992
Chr4	166929102	G	A	rs17590218	exonic	TLL1	NM_001204760:exon7:c.G819A<br>NM_012464:exon7:c.G819A	synonymous SNV	TLL1:NM_001204760:exon7:c.G819A:p.E273E<br>TLL1:NM_012464:exon7:c.G819A:p.E273E	0.0341	0.0521166
Chr4	166952032	T	G	rs141587160	UTR3	TLL1	NM_001204760:c.*407T>G			0	0.00299521
Chr4	166952084	T	C	rs72974358	UTR3	TLL1	NM_001204760:c.*459T>C			0	0.124201
Chr4	167012381	G	A	rs2654492	exonic	TLL1	NM_012464:exon19:c.G2544A	synonymous SNV	TLL1:NM_012464:exon19:c.G2544A:p.L848L	0.9009	0.90615
Chr4	167012480	T	C	rs2646914	exonic	TLL1	NM_012464:exon19:c.T2643C	synonymous SNV	TLL1:NM_012464:exon19:c.T2643C:p.A881A	0.9714	0.969848
Chr4	167020644	A	G	rs2291822	exonic	TLL1	NM_012464:exon20:c.A2872G	nonsynonymous SNV	TLL1:NM_012464:exon20:c.A2872G:p.T958A	0.0923	0.164736
Chr4	167023255	A	G	rs1353681	UTR3	TLL1	NM_012464:c.*1227A>G			0	0.19988
Chr4	167023504	T	G	rs1909084	UTR3	TLL1	NM_012464:c.*1476T>G			0	0.156949
Chr4	167023760	G	T	rs1909083	UTR3	TLL1	NM_012464:c.*1732G>T			0	0.164537
Chr4	167024479	C	T	rs1497950	UTR3	TLL1	NM_012464:c.*2451C>T			0	0.998802
Chr4	167024993	G	A	rs200203328	UTR3	TLL1	NM_012464:c.*2965G>A			0	0
Chr4	167025006	-	A	rs33999097	UTR3	TLL1	NM_012464:c.*2978_*2979insA			0	0.865815
Chr4	167025159	A	G	rs17633952	UTR3	TLL1	NM_012464:c.*3131A>G			0	0.201877
Chr5	172662024	T	C	rs2277923	exonic	NKX2-5	NM_001166175:exon1:c.A63G<br>NM_001166176:exon1:c.A63G<br>NM_004387:exon1:c.A63G	synonymous SNV	NKX2-5:NM_001166175:exon1:c.A63G:p.E21E<br>NKX2-5:NM_001166176:exon1:c.A63G:p.E21E<br>NKX2-5:NM_004387:exon1:c.A63G:p.E21E	0.4042	0.535743
Chr7	35293193	A	G	rs336283	exonic	TBX20	NM_001077653:exon1:c.T39C<br>NM_001166220:exon1:c.T39C	synonymous SNV	TBX20:NM_001077653:exon1:c.T39C:p.S13S<br>TBX20:NM_001166220:exon1:c.T39C:p.S13S	0.7463	0.69389
Chr7	35293417	A	G	rs73099190	UTR5	TBX20	NM_001077653:c.-186T>C<br>NM_001166220:c.-186T>C			0	0.33107
Chr8	11566151	C	T	0	exonic	GATA4	NM_002052:exon2:c.C330T	synonymous SNV	GATA4:NM_002052:exon2:c.C330T:p.F110F	0	0
Chr8	11566308	C	T	rs387906769	exonic	GATA4	NM_002052:exon2:c.C487T	nonsynonymous SNV	GATA4:NM_002052:exon2:c.C487T:p.P163S	0	0.00019968
Chr8	11616338	A	C	rs867858	UTR3	GATA4	NM_002052:c.*354A>C			0	0.361422
Chr8	11616516	T	C	rs904018	UTR3	GATA4	NM_002052:c.*532T>C			0	0.632188
Chr8	11616547	C	G	rs12825	UTR3	GATA4	NM_002052:c.*563C>G			0	0.455072
Chr8	11616571	A	G	rs804291	UTR3	GATA4	NM_002052:c.*587A>G			0	0.979433
Chr8	11617240	A	T	rs12458	UTR3	GATA4	NM_002052:c.*1256A>T			0	0.39996
Chr12	114791952	A	-	rs35534655	UTR3	TBX5	NM_000192:c.*1385delT<br>NM_181486:c.*1385delT<br>NM_080717:c.*1385delT			0	0.790136
Chr12	114792236	T	C	rs6489956	UTR3	TBX5	NM_000192:c.*1101A>G<br>NM_181486:c.*1101A>G<br>NM_080717:c.*1101A>G			0	0.808307
Chr12	114792953	A	G	rs12426660	UTR3	TBX5	NM_000192:c.*384T>C<br>NM_181486:c.*384T>C<br>NM_080717:c.*384T>C			0	0.043131
Chr14	23855314	C	T	rs77416370	exonic	MYH6	NM_002471:exon34:c.G4986A	synonymous SNV	MYH6:NM_002471:exon34:c.G4986A:p.A1662A	0.0002	0.00339457
Chr15	35080829	A	G	0	UTR3	ACTC1	NM_005159:c.*1784T>C			0	0
Chr15	35082225	C	T	rs1370154	UTR3	ACTC1	NM_005159:c.*388G>A			0	0.278554
Chr18	19780858	G	A	rs1941084	UTR3	GATA6	NM_005257:c.*72G>A			0	0.51857
